# Associations of specific-age and decade recall body mass index trajectories with obesity-related cancer

**DOI:** 10.1186/s12885-021-08226-4

**Published:** 2021-05-05

**Authors:** Charlotte Watson, Andrew G. Renehan, Nophar Geifman

**Affiliations:** 1 Manchester Cancer Research Centre and NIHR Manchester Biomedical Research Centre, Manchester, UK; 2 Division of Cancer Sciences, School of Medical Sciences, Faculty of Biology, Medicine and Health, University of Manchester, Manchester, UK; 3 Centre for Health Informatics, Division of Informatics, Imaging and Data Sciences, School of Health Sciences, Faculty of Biology, Medicine and Health, University of Manchester, Manchester, UK

**Keywords:** Body mass index, Cancer, Statistical learning

## Abstract

**Background:**

Excess body fatness, commonly approximated by a one-off determination of body mass index (BMI), is associated with increased risk of at least 13 cancers. Modelling of longitudinal BMI data may be more informative for incident cancer associations, e.g. using latent class trajectory modelling (LCTM) may offer advantages in capturing changes in patterns with time. Here, we evaluated the variation in cancer risk with LCTMs using specific age recall versus decade recall BMI.

**Methods:**

We obtained BMI profiles for participants from the Prostate, Lung, Colorectal and Ovarian Cancer Screening Trial. We developed gender-specific LCTMs using recall data from specific ages 20 and 50 years (72,513 M; 74,837 W); decade data from 30s to 70s (42,113 M; 47,352 W) and a combination of both (74,106 M, 76,245 W). Using an established methodological framework, we tested 1:7 classes for linear, quadratic, cubic and natural spline shapes, and modelled associations for obesity-related cancer (ORC) incidence using LCTM class membership.

**Results:**

Different models were selected depending on the data type used. In specific age recall trajectories, only the two heaviest classes were associated with increased risk of ORC. For the decade recall data, the shapes appeared skewed by outliers in the heavier classes but an increase in ORC risk was observed. In the combined models, at older ages the BMI values were more extreme.

**Conclusions:**

Specific age recall models supported the existing literature changes in BMI over time are associated with increased ORC risk. Modelling of decade recall data might yield spurious associations.

**Supplementary Information:**

The online version contains supplementary material available at 10.1186/s12885-021-08226-4.

## Background

Excess body fatness, commonly approximated by body mass index (BMI), is a risk factor for several cancer types. In 2016, the International Agency for Research on Cancer (IARC) reviewed the epidemiological literature concluding that there was sufficient evidence to link BMI with increased risk in at least 13 cancer types including colon and rectal, postmenopausal breast, endometrium, ovary, liver, gallbladder, pancreas, gastric cardia, kidney, thyroid cancers, oesophageal adenocarcinoma, meningioma and multiple myeloma [1]. There are biologically plausible mechanisms underpinning these associations [2]. However, the majority of this evidence is based on one-off determination of BMI, typically in middle adulthood. These may not be reflective of the long-term exposure of excess body fatness and fails to capture, for example, what the BMI value was entering adulthood (in turn, reflecting childhood excess body fatness), rates of changes in BMI, and when changes in BMI occurred in the life course.

Modelling of longitudinal BMI data may be more informative for incident cancer associations. There are various methods to do this ranging from simply averaging relative or absolute changes over time [3]; to capturing metrics of years of excess body fatness [4, 5] to more advanced modelling, such as k-mean clustering [6], mixed-effect modelling [7], and latent class trajectory models (LCTMs). This study utilises LCTM, a modelling approach that simplifies heterogeneous populations into more homogeneous clusters or classes over time. LCTMs allow for the inclusion of random effects to allow for individual variation within these classes [8]. These models are increasingly reported in cancer epidemiology and have been used in association studies of repeated BMI measures with the following endpoints: cancer incidence (multiple cancer types [9], gastro-oesophageal [10], prostate [11], and cancer mortality [9].

The approach of LCTM has the potential advantage of data-driven unsupervised modelling but is disadvantaged by measurement errors in repeated sampling of the exposure of interest. Specifically, for body fatness, BMI determinants are derived from recall. This is seldom validated. In turn, recall weight might be specific age (e.g. age 20 years [12]) or decade recall [13] (e.g. age in your 20s). To our knowledge, there is no study examining whether specific age recall BMI differs to decade recall measures. Here, we used the Prostate, Lung, Colorectal and Ovarian (PLCO) Cancer Screening Trial to evaluate the variation in cancer risk with LCTMs using specific age recall versus decade recall BMI.

## Methods

### Study population

Between 1993 and 2001, the PLCO study enrolled 154,897 participants (76,682 men, 78,215 women) from multiple cancer screening centres across the USA. The design of this study has been previously detailed [14]. Briefly, participants were randomized to an intervention or usual care arm. Those in the intervention arm received up to 6 annual cancer screening tests whereas those in the usual care arm followed standard procedures. A supplemental questionnaire (SQX) was added between 2006 and 2008 and mailed to both arms of the trial, 87% of the original cohort responded [15].

From the baseline PLCO cohort the initial age range at recruitment was 55–74, however as SQX was added several years later we removed those aged over 80 (6920 from the SQX dataset) as changes in weight could be due to muscle wastage or other aging related factors, those with no baseline BMI (7231 from baseline cohort, 3952 from SQX) and those with an implausible BMI outside the range of 15–60 kg/m^2^ (5 from SQX cohort).

### BMI ascertainment

Upon study entry participants were asked to provide current height and body weight, as well as recall these metrics for ages 20 and 50. BMI was then calculated for each time point (weight [kg] / height [m]^2^). As part of the SQX participants were asked to recall their weight when they were in their 30s, 40s, 50s, 60s and 70s, (“Please estimate your weight when you were [in your 30s]. (Exclude any periods when you were pregnant).”) as well as provide their current weight and height. BMI was then calculated for each decade. Therefore, for specific age recalls, a maximum of 3 BMI measures per participant were used (with a mean of 2.99), for decade recalls a maximum of 6 (mean of 5.48), and for the combined model, 9 potential BMI measures.

### Ascertainment of cancer incidence

Cancer incidence was monitored through annual follow up questionnaires until 2010 and cancers reported on these questionnaires underwent a confirmation process, in which relevant medical records were obtained to verify the diagnosis and ICD-0 code of the cancer. From 2011, participants were switched to follow up via passive linkage to cancer registries and the National Death Index [16].

To illustrate how different latent class assignment can affect the reported risk of an ORC, we determined incident risk of 12 IARC ORCs (listed in Supplementary Table 1, multiple myeloma was excluded as this diagnosis is not well-captured in ICD codes).

### LCTM development

Using the identified BMI measures, we derived LCTMs with ORC, as the outcome measure, separately in men and women, for specific age recall only (**Subset A**), decade recall only (**Subset B**) and all measures (**Subset C**). For the models in Subset C, a variable indicating whether the BMI measure was specific recall or decade recall was included as a covariate, as was an interaction term with that variable over time. This allowed the identification of which sets of data were driving the final trajectories, and how the final trajectories might change dependent on the proportion of each type of recall data included in each class. This model was then also used to determine any similar classes that arose from Subsets A or B.

Following the published framework for developing latent class trajectories [8], we developed a scoping model for each subset to determine the random effect structure (Supplementary Figure 2). Using these, cubic random effects were selected for all models.

For each subset, multiple trajectory shape structures and number of latent classes (up to k = 7) were tested. Twenty random start points were run to ensure that the model had reached its global maximum and the log-likelihoods plotted to ensure that the majority had converged on the same model.

As suggested by Nagin et al. [17] and other sources, we used multiple metrics to determine which model was the best suited to each subset of data. This included the average probability of assignment (APPA), odds of correct classification (OCC), relative entropy and the Bayesian Information Criterion (BIC). The best fitting models were then plotted to ensure clinically plausible patterns were observed. Model discrimination was assessed through Elsensohn’s envelope of residuals and degrees of separation between the derived classes.

For both men and women in all subsets, linear, quadratic, and cubic models were fitted for classes 1:7. In all models cubic k = 7 did not converge, justifying our cut off point for number of classes tested. Due to the data structure, a natural spline model was also fitted for **Subset B** with knots at 30s, 40s and 50s, and **Subset C** with knots at 40s,50s and 60s.

As more classes were added to the models, the BIC for each shape decreased so the “elbow” criterion was applied to determine the best fit, along with performance in other metrics. In **Subset A**, a cubic model with 5 classes was selected for both men and women. In **Subset B**, a 7-class natural spline model in men, and a 6-class natural spline model was chosen in women. Finally, for **Subset C** a 4-class natural spline model was chosen for men and a cubic 4 class model chosen for women.

Baseline characteristics are described for **Subset C** by final LCTM class in Supplementary Tables 2 and 3.

### Time to event analyses

To assess how each latent class was linked to ORC incidence and how difference in model selection could affect the reported incident risk, we fitted Cox proportional hazards models, with age in years as the time metric, adjusted for smoking status at baseline (current, former, never) and baseline hazards stratified by age category at study entry in five-year intervals. From this we estimated hazard ratios (HRs) and 95% confidence intervals (CI) of the association of each class to ORC.

As there are several confounding factors between obesity and cancer, a secondary Cox model was fitted to also include diabetic status, presence of a heart condition, educational level, race, family history of cancer, NSAID use (aspirin or ibuprofen), and in women Hormone Replacement Therapy (HRT) status.

All statistical analysis was undertaken in R (version 3.6.0) using the *lcmm* package to develop the LCTMs and the *LCTMtools* package to examine model performance.

### Sensitivity analysis

All final models were re-run with only participants assigned to each class with a posterior probability of > 80% to confirm that the model shapes remained stable when including only those participants with a higher class assignment certainty.

## Results

We included those with at least 2 BMI measures, resulting in a population of 147,350.

(72,513 M; 74,837 W) for **Subset A**, 89,465 (42,113 M; 47,352 W) for **Subset B** and 150,351 (74,106 M; 76,245 W) for **Subset C.** Subset C has the highest number as those who were excluded in Subset A or Subset B for only having one measure, but had one response in both the baseline and supplementary surveys could then be included in the combined model. The flow diagram is detailed in Supplementary Figure 1. A minimum of 2 data points was used as LCTMs are very adept at dealing with missing data, and this meant that the maximum amount of data could be included. (e.g. in Subset A, where if one data point was excluded all data for that individual would also otherwise be excluded) without allowing complete reliance on the model to predict the entire trajectory for that individual if only 1 data point was included.

### BMI trajectories derived from specific age recall data (subset a)

**Fig. 1:**
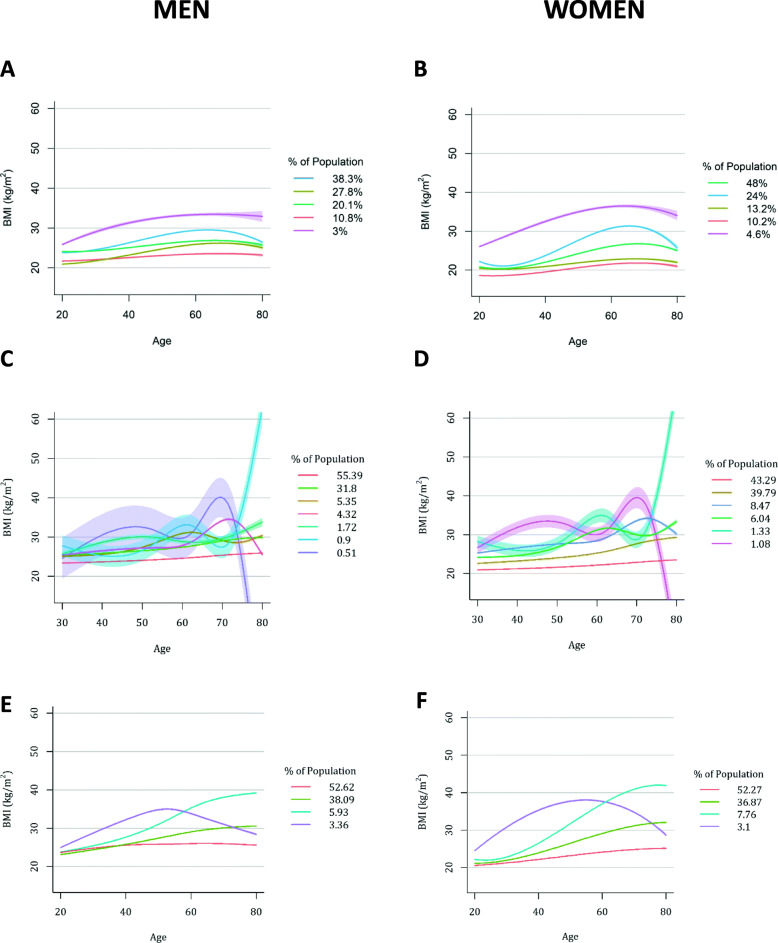
Derived BMI Trajectories in both men and women using differently collected BMI measures. Panels **a** and **b** show trajectories derived from specific age recall BMI measures, **c** and **d** show trajectories derived from decade recall BMI measures, and **e** and **f** show trajectories derived from all BMI data. All show a 95% confidence interval for participant assignment to each class

In subset A, cubic models with 5 classes were selected for both men and women. In men (Fig. 1a), the model comprised the following classes with time: “lean stable” starting and remaining normal weight over time; “lean increase”, starting normal weight and gaining weight; “medium stable” starting and remaining overweight; “medium increase”, starting overweight and gaining weight until 60s before losing weight by 80; and “heavy increase”, starting overweight and gaining weight until 40s and maintaining obese status.

In women (Fig. 1b), the best-fit model identified trajectories different than those in men. These comprised the following classes with time: “lean s shaped”, starting borderline underweight and gaining weight, then losing weight after 70 years of age; “lean small increase”, gaining weight but remaining normal weight; “lean moderate increase”, gaining weight and becoming overweight around 60s; “lean heavy s shaped”, gaining weight and becoming obese at 60 years of age, then losing weight to become borderline overweight/normal weight by 80 years of age; and “heavy increase”, similar to men.

### BMI trajectories derived from decade recall (subset B)

In subset B analyses, seven classes were identified in men, and six identified in women, both in a splenic shape. In men (Fig. 1c), we identified the following classes over time:

“lean increase” and a “medium s shaped”, who gained weight becoming obese by 50s, then losing weight into the overweight category, only to gain weight again around 70s; “medium increase”, who gained weight over time, and a “medium delayed n shaped” class, who remained overweight until 60s, gained weight and subsequently lost it; a new “double peak” class, gaining weight until 30s, losing it, then gaining more weight by 60s, peaking at morbidly obese before rapid weight loss; “heavy s-shaped”, gaining weight to become obese by 40s, losing some weight and then re-gaining weight after 60s; and “heavy s shaped increase”, gaining weight from 40s, losing it by 60s, and rapidly re-gaining.

In women (Fig. 1d), some similar classes emerged, with the “lean increase”, “medium increase”, “medium delayed n shaped”, “heavy s shaped increase”, “double peak”, “heavy s-shaped” showing similarities to those seen in men. However, the gradients of these classes and proportion of the population assigned to each do differ to the derived model in men.

### BMI trajectories derived from all measurements recall data (subset C)

We ran the same trajectory models on datasets comprising of both age-specific recall data and decade recall data. In men (Fig. 1e), we identified four splenic shaped classes over time: “high lean stable” who were borderline overweight constantly, “lean-heavy increase”, starting normal weight but rapidly gaining weight becoming overweight by 40s and eventually obese in later life; “medium heavy increase”, gaining weight at a fast rate becoming morbidly obese and “n-shaped”, gaining weight to become morbidly obese by 50s and then losing weight but remaining borderline obese.

In women (Fig. 1f), we identified four cubic shaped classes over time: “lean moderate increase”, gaining weight to become overweight; “lean heavy increase” gaining weight at a high rate to became obese; “lean extreme increase”, gaining weight at a rapid rate to become obese by 50s and morbidly obese by 60s; and “n shaped”, gaining weight until 50s, then losing weight.

### Associations between classes and ORC incidence

To assess the relationships between the various identified trajectories of BMI and the risk of obesity related cancer, we examined incidence of such cancers with trajectory groups identified by each of our models. Overall, we found that across all subsets, a stepwise increase in ORC incidence was observed with increasing BMI trajectory. When non-ORCs where examined, no significant associations were found (Supplementary Figure 3).

In specific age recall trajectories in men, those that were lean when younger (“lean increase” group) or remained a constant weight (“medium stable”) had a no significant increase in.

**Fig. 2:**
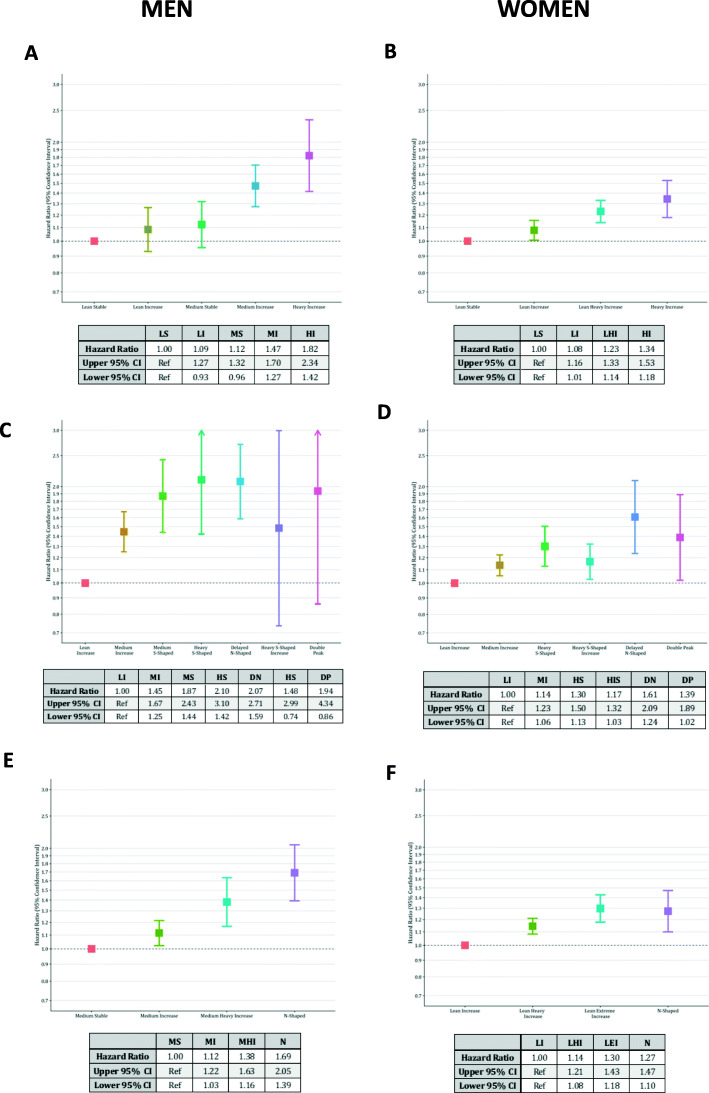
Time-to-event analysis for each derived trajectory class in men and women. Age specific trajectories (**a** and **b**), decade recall trajectories (**c** and **d**) and all measures trajectories (**e** and **f**). All models adjusted for smoking status (current, former, never) and stratified by age category at study entry (5-year groups)

ORC risk relative to those in the “lean stable” group. However, those that started as overweight and gained weight over time had an increased risk (HR: 1.47 & 1.82 respectively) as shown in Fig. 2a. This was also seen in the men’s model for decade recall in Fig. 2c, where all classes heavier than the referent group were associated with an increased ORC risk. However, here the heavier classes have very large confidence intervals, and the association is not significant. Finally, in the combined model (Fig. 2e), a similar association was observed with all classes, with the greatest risk being seen in those that gained weight at the fastest rate (HR: 1.69, 95% CI: 1.39–2.05).

For women, a similar pattern is seen. In the specific age recall model, again the two heavier classes have a significant increase in ORC risk (HR: 1.23 & 1.34), although this is weaker than in men for the heaviest group (HR:1.34 vs 1.82 in men) (Fig. 2b). For decade recall, there was again a stepwise increase in risk, although at a lower level than observed in men (Fig. 2d). The confidence intervals are still larger than the specific age recall data, but not as large as those seen in men. The combined model exhibits the same pattern as seen in the specific age recall with a stronger association and slightly increased confidence intervals, indicating extra noise incorporated into the model (Fig. 2f).

### Comparison between specific age recall and decade recall data

**Fig. 3:**
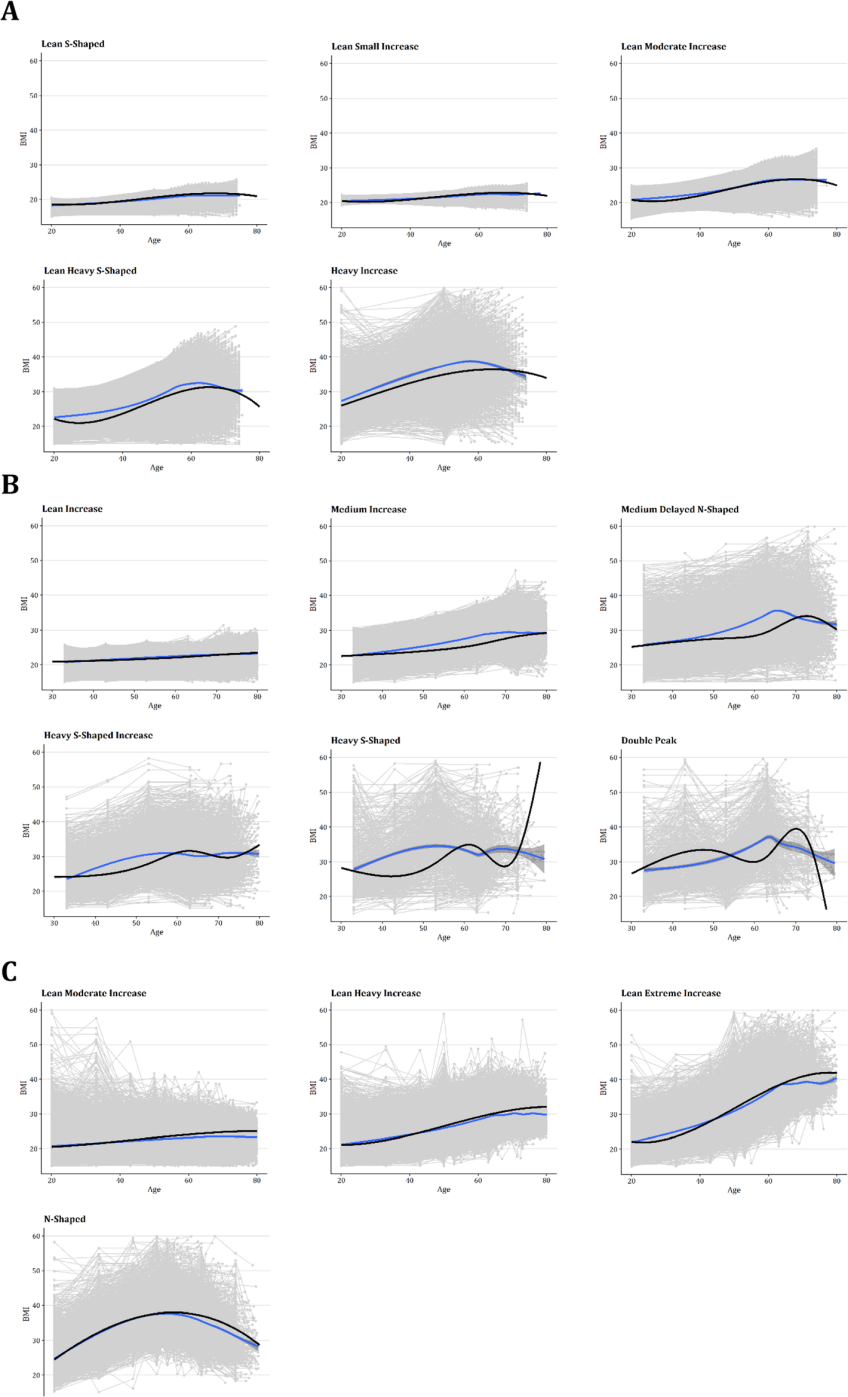
Comparison of raw data class assignment vs LCTM predicted trajectory. Specific age recall model (**a**), decade recall model (**b**) and combined model (**c**). Raw data for each participant assigned to the class shown in grey, with a smoothing spline fit in blue with a 95% confidence interval. Predicted trajectory from the model shown in black

To compare how the data differed between specific age recall and decade recall and how this affected the fitted models, the raw data assigned to each class and the predicted trajectory were plotted to assess goodness of fit. This is shown for women in Fig. 3 (men in Supplementary Figure 4). Here Fig. 3a represents the 5-class cubic model fitted for the specific age recall data, 3B for the decade recall data and 3C for the combined data model. As shown, the mean of the raw data (dark blue) at each recalled time point vs the model trajectories align well for both the specific age and combined models. However, in the decade recall trajectories, the model constantly underfits the raw data, and in the heavier more extreme classes, predicts the opposite direction of weight change. This pattern was observed in several tested models for this data subset, with varying class numbers and shapes, indicating that the model choice is not the root cause of this issue.

## Discussion

### Main findings

Our results indicate that decade age recall data introduces noise into the trajectories, with larger confidence intervals observed both in trajectory assignment and reported ORC risk. We hypothesised that six decade recall measures might be more informative than three age specific recall points, but this did not transpire to be the case. This raises a note of caution for others using unsupervised machine learning methods to cluster data, that probabilistic methods such as LCTMs can be easily skewed.

Secondly, the use of the decade recall data, for which more data points were available, resulted in trajectories that were clinically implausible and showed changes in BMI that were unrealistic. This is interesting as the original hypothesis was that additional time points and data would allow for more complex trends to be captured, but also reduce the potential effect of noise. Nevertheless, this may also increase the risk of overfitting. Finally, when the latent classes were tested against ORC and non-ORC incidence, increased risk for ORC was observed for the heavier trajectories, in all models, but with varying confidence. There were no significant results for the non-obesity related cancers. Here it was seen that for the decade recall models, the confidence intervals were large, and a clear pattern in risk was not as easily observed as the specific age models. Again, this is likely due to the wide confidence intervals for class assignment in the model and implies that more noise is being introduced in the decade recall models.

### Comparisons with other studies

To our knowledge, this is the first study to examine the differences in BMI trajectories derived from participants’ recall of their weight at a specific time point vs a rounded (decade) time point. Although previous studies have looked at the effects of the age at which recall weight data is collected, these have not been examined in the context of trajectory modelling.

It has been shown that while recalled weight is close to the average of true weight in a population, per person there can be large variation. This can be affected by a person’s current weight, any past changes in weight and current cognitive ability. For example, those that were at the extreme ends of the spectrum (underweight, obese) tended to normalise their results by over/under estimating their previous weight [18].

LCTMs have previously been derived in the PLCO cohort in men by Kelly et al. [11], however this was only done on the specific age recall data. Here they derived 5 classes in men, compared to our six, this is likely due to the use of specific age recall data only. When compared to our combined model, we observed similarities for the three largest classes. Our “lean stable” class followed the same path as their “stable normal” class and had almost identical proportions of the population (33% vs 34%). The “lean increase” group we derived, was similar in trajectory to the “normal-overweight” group although ours was slightly smaller (39% vs 47%). Finally, our “medium increase” group appeared to be similar to the “stable overweight” group, although ours started at a lower BMI. The final two classes in our own model and that described by Kelly et al., are the smallest and differences could be due to differences in model specifications and different sample sizes (as we did not exclude those with a prior history of cancer from the trajectory cohort, only when deriving time to event hazard ratios).

Another study pooled the PLCO cohort with the NIH-AARP cohort in order to derive BMI trajectories based on the specific-age recall measures [10]. Although the resulting trajectories were not assessed separately in men and women, similarities between the largest three groups, with our own results were seen. These included a group that remains at a normal BMI over life course, a group that starts at a normal BMI and become overweight, and a group that start as overweight and become obese.

When using decade recall data to determine incident ORC risk, we had similar findings to Lu et al. [13]. They showed that those who put on weight in their 20s had a higher risk than those who gained weight later on in life (50s/60s). In our models, the BMI recorded in the 30s appears to have the most influence over ORC risk, as the reported risk shows a stepwise increase across BMI class.

### Strengths and limitations

There were a number of strengths to this study. Firstly, the cohort used is well-documented and has a large sample size. Secondly, by deriving and selecting our models following the published framework [8], we ensured that the model best fit the data. Thirdly by using lots of sensitivity analyses we determined that models we had derived were robust and did not contain spurious associations. Finally, we made good use of the richness of the data, with up to 9 recalled BMI data points per person used in the final combined model.

However, this study does have limited generalisability as the majority of the population were white and well educated. As most measures (excluding baseline) were recalled this could have led to some bias for extreme weight groups, although similar cohorts have mostly found good correlation between self-reported and directly measured anthropometric data. Finally, due to computational constraints not all models could be run. For example, to be comparable with other latent class studies in the literature, testing up to 10 classes would have been preferable, but we were restricted to 7 due to computational power restrictions. However, the elbow bend of the BIC indicated that for example in our specific age recall models, 4/5 classes were optimum, so it is unlikely that classes 8–10 would have resulted in better fitted models. It was also reassuring to see similar classes emerging in each set of data, showing that these are more likely “true” classes.

### Unanswered questions and future research

Further work is needed to determine whether the results generated above are study-specific or generalisable to other datasets. Future work will be to derive these models in other cohorts and determine whether the resulting trajectories are similar. Testing multiple data sources with diverse participants would likely bring out new classes and characteristics that are not seen in predominantly white cohorts.

In addition, there is no clear answer as to why the decade recall data is so noisy, as any outliers were removed, and all data fell within a reasonable range. Further work is required to assess the usability and reliability of these and similarly noisy data, specifically for trajectory modelling.

## Conclusions

Here we show that decade recall data introduces more noise into the model than just using specific age recall. However, it is hard to distinguish if this is due to data quality or just the addition of more data points (up to 6-decade recall measures vs 3 specific age measures). This can have an impact on reported relationship with time-to-event outcomes and great care should be taken with interpretation of these.

Overall, we have shown that there is evidence to suggest that decade recall data does not give the same clarity in modelling as specific age recall data, and would recommend that researchers take into account how their data is collected when interpreting the models, to prevent over-reliance on a trajectory that could be due to an inaccurate noisy training set.

## Supplementary Information

**Additional file 1: Table S1.** Obesity Related Cancers. **Table S2.** Baseline Characteristics for Women by latent class assignment. **Table S3.** Baseline Characteristics for Men by latent class assignment. **Figure S1.** PLCO data exclusions. **Figure S2.** Scoping model for men and women. **Figure S3.** Non obesity related cancer incidence for the combined data trajectories. **Figure S4.** Raw trajectories vs Predicted Trajectory in men.

## Data Availability

The data that support the findings of this study are available from the NCI, but restrictions apply to the availability of these data, which were used under license for the current study, and so are not publicly available. Data are however available from the authors upon reasonable request and with permission of NCI.

## Abbreviations

BIC: Bayesian Information Criterion
BMI: Body Mass Index
HRT: Hormone Replacement Therapy
IARC: International Agency for Research on Cancer
LCTM: Latent Class Trajectory Model
ORC: Obesity Related Cancer
PLCO: Prostate, Lung, Colorectal and Ovarian Cancer Screening Trial
