# Supplementary material for: Associations of specific-age and decade recall body mass index trajectories with obesity-related cancer
Source: BMC Cancer. 2021 May 5;21:502. doi: 10.1186/s12885-021-08226-4 (PMC8097878; doi:10.1186/s12885-021-08226-4)
Supplement: Supplementary file 1 — Additional file 1: Table S1. Obesity Related Cancers. Table S2. Baseline Characteristics for Women by latent class assignment. Table S3. Baseline Characteristics for Men by latent class assignment. Figure S1. PLCO data exclusions. Figure S2. Scoping model for men and women. Figure S3. Non obesity related cancer incidence for the combined data trajectories. Figure S4. Raw trajectories vs Predicted Trajectory in men. [file 12885_2021_8226_MOESM1_ESM.docx]

**Supplementary material**

**A latent class trajectory comparison of specific age and decade recall in the context of obesity related cancer**

Charlotte Watson, Professor Andrew G Renehan and Dr Nophar Geifman

**Supplementary Table 1: Obesity Related Cancers**

| **Cancer Site** | **ICD-10 code** |
| --- | --- |
| Oesophagus (*lower third*) | C15.5, C15.8 |
| Colorectal | C18.0 - 18.9, C19.9, C20.0 |
| Liver | C22.0 |
| Gallbladder | C23.9 |
| Pancreas | C25.0 - 25.9 |
| Breast* | C50.0 - 50.9 |
| Corpus Uteri / Endometrial | C54.0 - 54.9, C55.9 |
| Ovary | C56.9 |
| Kidney | C64.9 |
| Gastric Cardia | C16.0 |
| Malignant meningioma | C70.0, C70.1, C70.9 |
| Thyroid | C73.0, C73.9 |
| Multiple myeloma | C90.0 |

** Breast was not stratified by menopausal status*

Lauby-Secretan B, Scoccianti C, Loomis D, Grosse Y, Bianchini F, Straif K. Body Fatness and Cancer--Viewpoint of the IARC Working Group. Colditz G, Anderson AS, Herbert RA, Kaaks R, Thompson HJ, Baker JL, Breda J, Byers T, Cleary MP, Di Cesare M, Gapstur SM, Gunter M, Hursting SD, Leitzmann M, Ligibel J, Renehan A, Romieu I, Shimokawa I, Ulrich CM, Wade K, Weiderpass E. *N Engl J Med* 2016; **375:** 794-8.

**Supplementary Table 2: Baseline Characteristics for Women by latent class assignment**

|  | **Lean Small Increase** | **Lean Moderate Increase** | **Lean Heavy Increase** | **Medium Increase** | **N-Shaped** |
| --- | --- | --- | --- | --- | --- |
| No of participants | 29134 | 74524 | 69314 | 37424 | 8531 |
| Mean entry age, years | 63.36 (5.47) | 63.23 (5.47) | 62.41 (5.3) | 61.32 (5.1) | 60.14 (4.73) |
| **Body Measures** | | | | | |
| Mean BMI at baseline, kg/m² | 20.71 (1.57) | 24.07 (1.75) | 28.41 (2.45) | 33.48 (4.75) | 37.73 (8.71) |
| Mean BMI at 20, kg/m² | 19.19 (1.51) | 20.3 (1.59) | 21.29 (1.81) | 23.31 (2.99) | 27.36 (6.31) |
| Mean BMI at 50, kg/m² | 20.19 (1.25) | 22.77 (1.43) | 25.77 (2.01) | 30.23 (4.09) | 36.43 (8.37) |
| **Comorbidities** | | | | | |
| Diabetes | 585 (2%) | 2164 (3%) | 4007 (6%) | 5048 (13%) | 2042 (24%) |
| Heart disease | 1141 (4%) | 3054 (4%) | 3153 (5%) | 2409 (6%) | 703 (8%) |
| **Smoking Status** | | | | | |
| Never | 15567 (53%) | 41519 (56%) | 39354 (57%) | 20959 (56%) | 4502 (53%) |
| Former | 9676 (33%) | 25735 (35%) | 24103 (35%) | 13301 (36%) | 3044 (36%) |
| Current | 3891 (13%) | 7258 (10%) | 5857 (8%) | 3161 (8%) | 985 (12%) |
| Missing | 0 (<1%) | 12 (<1%) | 0 (<1%) | 3 (<1%) | 0 (<1%) |
| **Ethnicity** | | | | | |
| Non Hispanic White | 25597 (88%) | 67678 (91%) | 62020 (89%) | 32141 (86%) | 7003 (82%) |
| Black | 605 (2%) | 2445 (3%) | 4251 (6%) | 3707 (10%) | 1151 (13%) |
| Hispanic | 355 (1%) | 1067 (1%) | 1102 (2%) | 716 (2%) | 169 (2%) |
| Asian | 2395 (8%) | 2887 (4%) | 1414 (2%) | 444 (1%) | 74 (1%) |
| Other | 170 (1%) | 429 (1%) | 503 (1%) | 401 (1%) | 125 (1%) |
| Missing | 12 (<1%) | 18 (<1%) | 24 (<1%) | 15 (<1%) | 9 (<1%) |
| **Highest Educational level achieved, %** | | | | | |
| <8 years | 125 (<1%) | 307 (<1%) | 501 (1%) | 455 (1%) | 128 (2%) |
| 8-11 years | 1143 (4%) | 3516 (5%) | 4077 (6%) | 2947 (8%) | 854 (10%) |
| Completed high school | 6713 (23%) | 19617 (26%) | 20521 (30%) | 11038 (29%) | 2273 (27%) |
| Post high school or college | 10228 (35%) | 26593 (36%) | 24962 (36%) | 13911 (37%) | 3353 (39%) |
| College/ Postgraduate | 10866 (37%) | 24329 (33%) | 19125 (28%) | 9023 (24%) | 1902 (22%) |
| Missing | 59 (<1%) | 162 (<1%) | 128 (<1%) | 50 (<1%) | 21 (<1%) |
| **Drug Use** | | | | | |
| Ibuprofen use 3+ times a week | 4705 (16%) | 12998 (17%) | 12868 (19%) | 6704 (18%) | 1459 (17%) |
| Missing | 164 (1%) | 498 (1%) | 449 (1%) | 329 (1%) | 95 (1%) |
| Aspirin use 3+ times a week | 5126 (18%) | 13034 (17%) | 12244 (18%) | 5925 (16%) | 1206 (14%) |
| Missing | 83 (<1%) | 220 (<1%) | 161 (<1%) | 123 (<1%) | 56 (1%) |
| HRT use ever, % | 20581 (71%) | 51263 (69%) | 45625 (66%) | 22682 (61%) | 4616 (54%) |
| Never | 8382 (29%) | 22860 (31%) | 23268 (34%) | 14459 (39%) | 3838 (45%) |
| **Outcomes** | | | | | |
| Number of cancer diagnoses, % | 3687 (13%) | 9340 (13%) | 8964 (13%) | 4739 (13%) | 1104 (13%) |
| Number of obesity related cancer diagnoses, % | 2051 (7%) | 5687 (8%) | 5708 (8%) | 3083 (8%) | 704 (8%) |
| Number of non-obesity related cancer diagnoses, % | 1564 (5%) | 3425 (5%) | 3056 (4%) | 1492 (4%) | 346 (4%) |
| Mean age of cancer diagnosis, y | 69.9 (6.51) | 70.04 (6.49) | 69.26 (6.33) | 68.04 (6.05) | 66.1 (5.89) |
| Mean age of obesity related cancer diagnosis, y | 69.21 (6.42) | 69.71 (6.51) | 68.79 (6.29) | 67.95 (5.96) | 65.72 (5.88) |
| Mean age of non-obesity related cancer diagnosis, y | 70.91 (6.5) | 70.66 (6.4) | 70.37 (6.24) | 68.4 (6.25) | 67.15 (5.89) |

**Supplementary Table 3: Baseline Characteristics for Men by latent class assignment**

|  | **Lean Increase** | **Lean Heavy Increase** | **High Lean Stable** | **Medium Increase** | **Medium Heavy Increase** | **N-Shaped** |
| --- | --- | --- | --- | --- | --- | --- |
| No of participants | 27947 | 34183 | 44549 | 42241 | 38772 | 2618 |
| Mean entry age, years | 63.89 (5.49) | 62.88 (5.34) | 63.36 (5.39) | 62.21 (5.16) | 61.39 (4.98) | 59.45 (4.54) |
| **Body Measures** | | | | | | |
| Mean BMI at baseline, kg/m² | 22.76 (1.69) | 27.31 (1.94) | 25.22 (1.43) | 28.93 (1.92) | 32.04 (4.5) | 35.6 (8.77) |
| Mean BMI at 20, kg/m² | 20.27 (1.49) | 20.41 (1.42) | 23.04 (1.29) | 24.76 (1.55) | 24.91 (3.74) | 27.18 (6.62) |
| Mean BMI at 50, kg/m² | 22.3 (1.43) | 25.46 (1.75) | 24.91 (1.25) | 28.03 (1.64) | 30.66 (3.82) | 35.05 (8.45) |
| **Comorbidities** | | | | | | |
| Diabetes | 1227 (4%) | 2670 (8%) | 2365 (5%) | 3690 (9%) | 7005 (18%) | 719 (27%) |
| Heart disease | 2966 (11%) | 4640 (14%) | 5310 (12%) | 5887 (14%) | 6502 (17%) | 500 (19%) |
| **Smoking Status** | | | | | | |
| Never | 10364 (37%) | 10924 (32%) | 17936 (40%) | 16032 (38%) | 12285 (32%) | 801 (31%) |
| Former | 12927 (46%) | 19714 (58%) | 21135 (47%) | 21882 (52%) | 22044 (57%) | 1439 (55%) |
| Current | 4656 (17%) | 3542 (10%) | 5469 (12%) | 4321 (10%) | 4437 (11%) | 375 (14%) |
| Missing | 0 (<1%) | 3 (<1%) | 9 (<1%) | 6 (<1%) | 6 (<1%) | 3 (<1%) |
| **Ethnicity** | | | | | | |
| Non Hispanic White | 23252 (83%) | 29782 (87%) | 39767 (89%) | 38676 (92%) | 34465 (89%) | 2276 (87%) |
| Black | 1346 (5%) | 1460 (4%) | 1470 (3%) | 1570 (4%) | 2146 (6%) | 162 (6%) |
| Hispanic | 572 (2%) | 927 (3%) | 886 (2%) | 775 (2%) | 947 (2%) | 80 (3%) |
| Asian | 2637 (9%) | 1682 (5%) | 2145 (5%) | 799 (2%) | 660 (2%) | 38 (1%) |
| Other | 134 (<1%) | 314 (1%) | 272 (1%) | 392 (1%) | 506 (1%) | 59 (2%) |
| Missing | 6 (<1%) | 18 (<1%) | 9 (<1%) | 29 (<1%) | 48 (<1%) | 3 (<1%) |
| **Highest Educational level achieved, %** | | | | | | |
| <8 years | 382 (1%) | 435 (1%) | 393 (1%) | 429 (1%) | 606 (2%) | 78 (3%) |
| 8-11 years | 1880 (7%) | 2700 (8%) | 2664 (6%) | 2547 (6%) | 3196 (8%) | 248 (9%) |
| Completed high school | 4514 (16%) | 6618 (19%) | 7262 (16%) | 7842 (19%) | 7982 (21%) | 542 (21%) |
| Post high school or college | 8385 (30%) | 11862 (35%) | 13348 (30%) | 13905 (33%) | 13998 (36%) | 966 (37%) |
| College/ Postgraduate | 12746 (46%) | 12467 (36%) | 20787 (47%) | 17425 (41%) | 12933 (33%) | 776 (30%) |
| Missing | 40 (<1%) | 101 (<1%) | 95 (<1%) | 93 (<1%) | 57 (<1%) | 8 (<1%) |
| **Drug Use** | | | | | | |
| Ibuprofen use 3+ times a week | 3491 (12%) | 5081 (15%) | 5904 (13%) | 6296 (15%) | 5826 (15%) | 429 (16%) |
| Missing | 68 (<1%) | 145 (<1%) | 146 (<1%) | 176 (<1%) | 198 (1%) | 12 (<1%) |
| Aspirin use 3+ times a week | 5460 (20%) | 6519 (19%) | 8917 (20%) | 8277 (20%) | 6861 (18%) | 380 (15%) |
| Missing | 48 (<1%) | 89 (<1%) | 72 (<1%) | 86 (<1%) | 78 (<1%) | 12 (<1%) |
| **Outcomes** | | | | | | |
| Number of cancer diagnoses, % | 3506 (13%) | 4096 (12%) | 5717 (13%) | 5064 (12%) | 4827 (12%) | 262 (10%) |
| Number of obesity related cancer diagnoses, % | 974 (3%) | 1161 (3%) | 1558 (3%) | 1602 (4%) | 1731 (4%) | 95 (4%) |
| Number of non-obesity related cancer diagnoses, % | 2520 (9%) | 2920 (9%) | 4144 (9%) | 3444 (8%) | 3075 (8%) | 167 (6%) |
| Mean age of cancer diagnosis, y | 71.09 (6.15) | 70.51 (6.23) | 70.96 (6.19) | 69.84 (6.28) | 68.76 (5.89) | 68.27 (5.93) |
| Mean age of obesity related cancer diagnosis, y | 70.47 (6.46) | 70.27 (6.29) | 70.72 (6.36) | 69.65 (6.31) | 68.81 (5.88) | 67.45 (5.91) |
| Mean age of non-obesity related cancer diagnosis, y | 71.35 (5.99) | 70.6 (6.19) | 71.06 (6.12) | 69.91 (6.26) | 68.7 (5.9) | 68.74 (5.9) |

**Supplementary Figure 1: PLCO data exclusions**

**
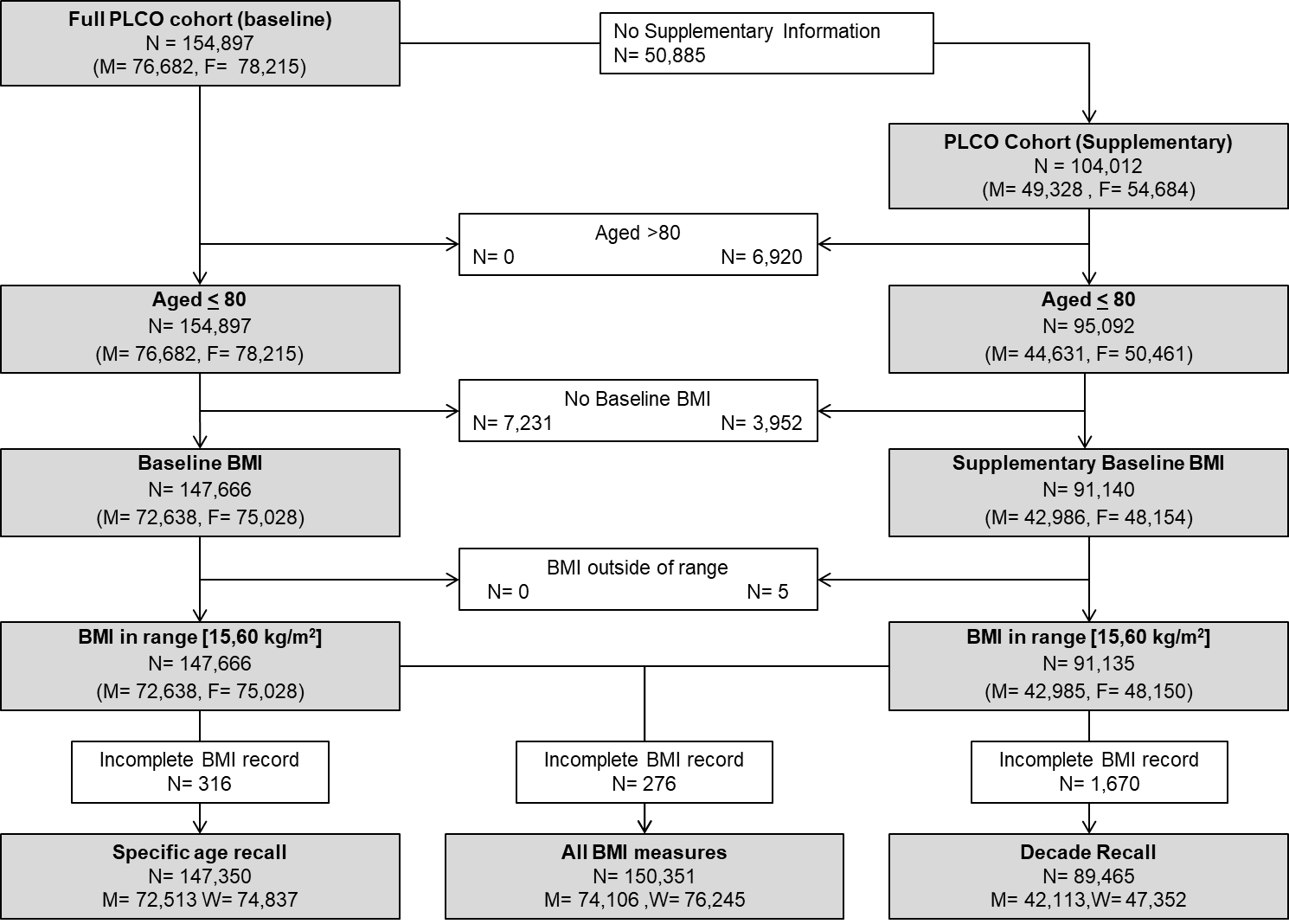
**

**
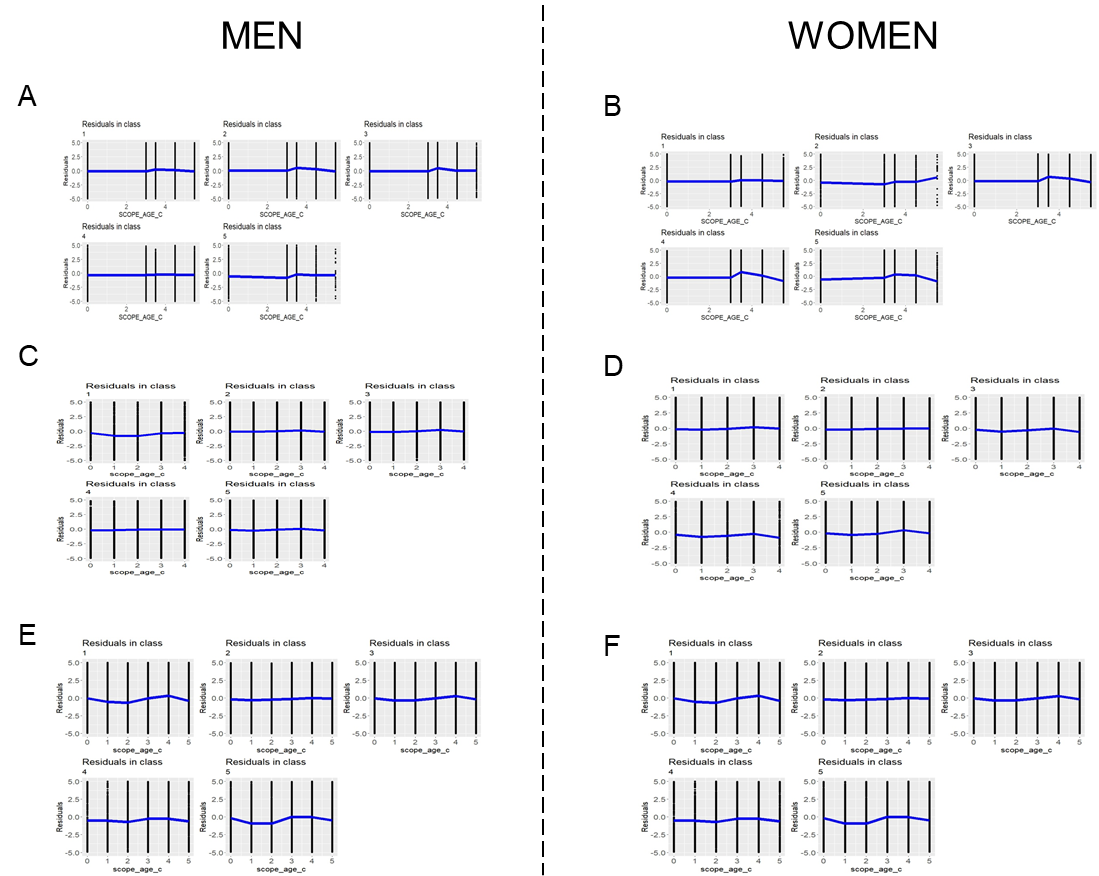
Supplementary Figure 2: Scoping model for men and women**

Panels A and B depict the residuals for the age specific recall data. C and D for decade recall data and E and F for all BMI data. Based on these, a cubic random effect structure was chosen for all.


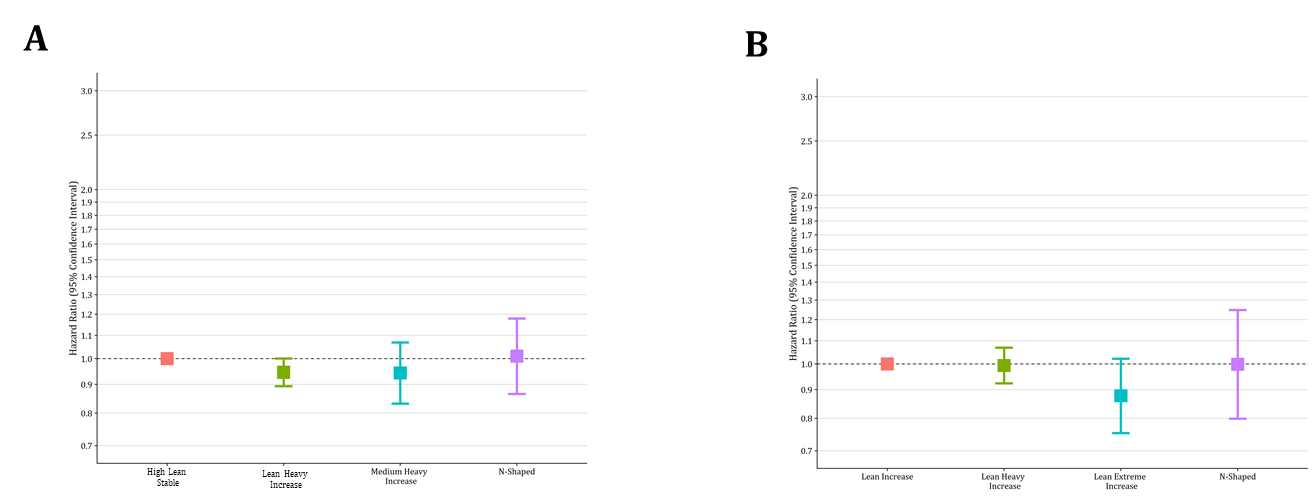
**Supplementary Figure 3: Non obesity related cancer incidence for the combined data trajectories**

Panel A shows the association of the “all data” trajectory class assignments in men with non-obesity related cancers. Panel B in women.

**
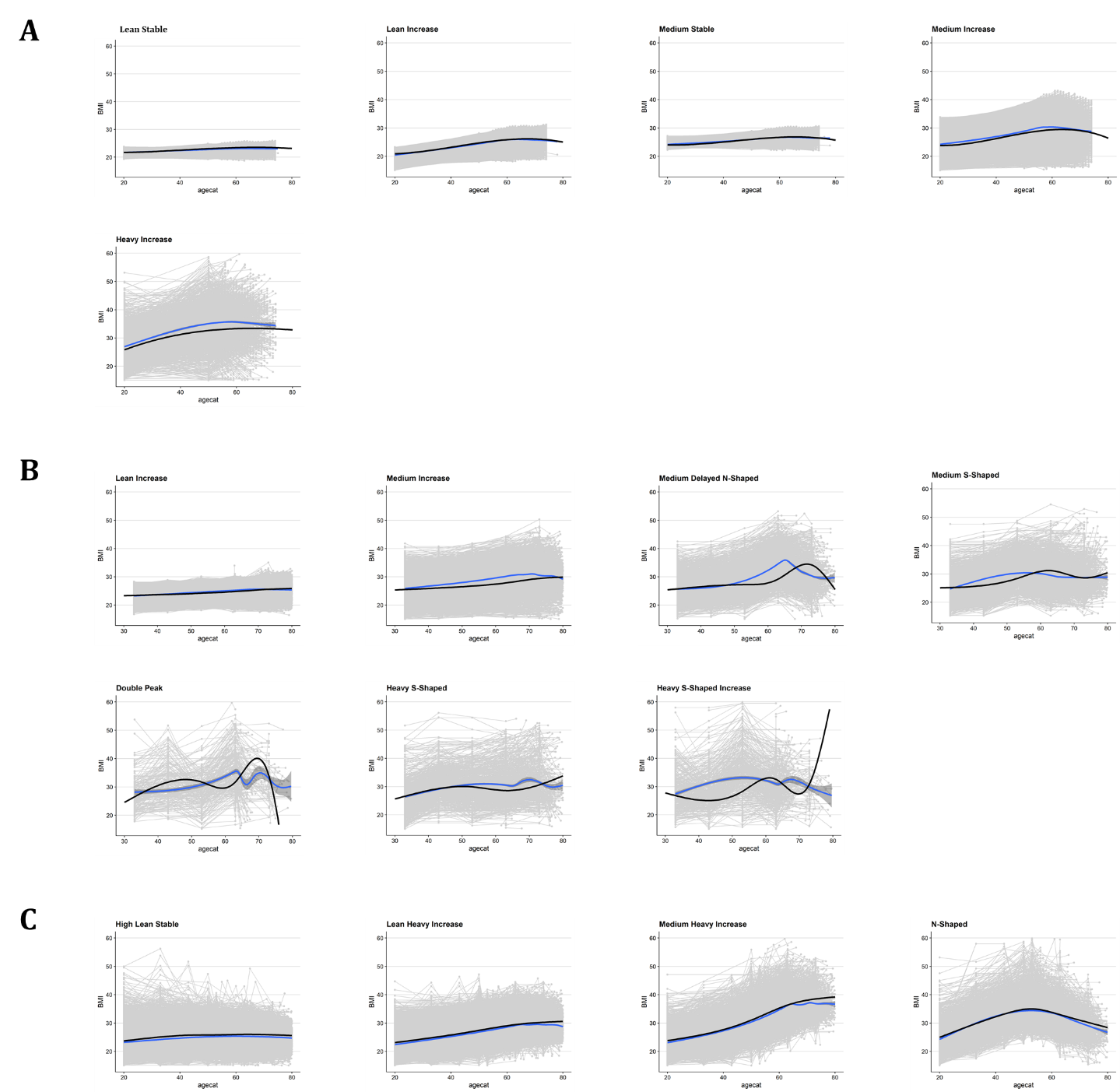
Supplementary Figure 4: Raw trajectories vs Predicted Trajectory in men**

Panel A depicts the difference between the raw data and the predicted trajectory for the model derived in specific age recalled data, Panel B for decade recalled data and Panel C for the combined data.
